# Elevated temperature alters bacterial community from mutualism to antagonism with *Skeletonema costatum*: insights into the role of a novel species, *Tamlana* sp. MS1

**DOI:** 10.1128/msphere.00198-24

**Published:** 2024-06-28

**Authors:** Tenghui Lin, Yumeng Feng, Wenfei Miao, Shuqi Wang, Zhen Bao, Zeyuan Shao, Demin Zhang, Xinwei Wang, Haibo Jiang, Huajun Zhang

**Affiliations:** 1 State Key Laboratory for Managing Biotic and Chemical Threats to the Quality and Safety of Agro-products, Ningbo University, Ningbo, China; 2 School of Marine Sciences, Ningbo University, Ningbo, China; 3 Key Laboratory of Applied Marine Biotechnology of Department of Education, Ningbo University, Ningbo, China; University of Wisconsin-Madison, Madison, Wisconsin, USA

**Keywords:** *Skeletonema costatum*, *Tamlana *sp. MS1, bacteria community, temperature elevation, interaction alteration

## Abstract

*Skeletonema costatum*, a cosmopolitan diatom primarily inhabiting coastal ecosystems, exhibits a typically close yet variable relationship with heterotrophic bacteria. The increasing temperature of surface seawater is expected to substantially affect the viability and ecological dynamics of *S. costatum*, potentially altering its relationship with bacteria. However, it remains unclear to what extent the elevated temperature could change these relationships. Here, the relationship between axenic *S. costatum* and natural seawater bacteria underwent a dramatic shift from mutualism to antagonism as the co-culture temperature increased from 20°C to 25°C. The co-occurrence network indicated significantly increased complexity of interaction between *S. costatum* and bacteria community after temperature elevation, especially with *Flavobacteriaceae*, implying their potential role in eliminating *S. costatum* under higher temperatures. Additionally, a *Flavobacteriaceae* isolate, namely MS1 identified as *Tamlana* genus, was isolated from the co-culture system at 25°C. MS1 had a remarkable ability to eliminate *S. costatum*, with the mortality rate at 25°C steadily rising from 30.2% at 48 h to 92.4% at 120 h. However, it promoted algal growth to some extent at 20°C. These results demonstrated that increased temperature promotes MS1 shifts from mutualism to antagonism with *S. costatum*. According to the comparative genomics analysis, changes in the lifestyle of MS1 were attributed to the increased gliding motility and attachment of MS1 under elevated temperature, enabling it to exert an algicidal effect through direct contact with alga. This investigation provided an advanced understanding of interactions between phytoplankton and bacteria in future warming oceanic ecosystems.

**IMPORTANCE:**

Ocean warming profoundly influences the growth and metabolism of phytoplankton and bacteria, thereby significantly reshaping their interactions. Previous studies have shown that warming can change bacterial lifestyle from mutualism to antagonism with phytoplankton, but the underlying mechanism remains unclear. In this study, we found that high temperature promotes *Tamlana* sp. MS1 adhesion to *Skeletonema costatum*, leading to algal lysis through direct contact, demonstrating a transition in lifestyle from mutualism to antagonism with increasing temperature. Furthermore, the gliding motility of MS1 appears to be pivotal in mediating the transition of its lifestyle. These findings not only advance our understanding of the phytoplankton-bacteria relationship under ocean warming but also offer valuable insights for predicting the impact of warming on phytoplankton carbon sequestration.

## INTRODUCTION

Ocean warming poses a significant threat to the stability of marine ecosystems. According to climate models, by the end of this century, surface seawater temperature is expected to increase as high as 6.4°C (1). This warming trend is critically involved in altering the structure of marine food webs and the biogeochemical cycles of the marine ecosystem (2, 3). Over the past century, it has been observed that the global decline of phytoplankton diversity is closely associated with the rise in surface seawater temperatures (4). The impact of ocean warming on phytoplankton is expected, as temperature exerts indirect effect by affecting stratification and nutrient flux (5) and direct effect by altering community composition and metabolic rates (6). Considering the pivotal role of phytoplankton in marine food webs, the impact of elevated temperatures on phytoplankton has gained significant attention.

Diatoms, constituting 20% of the global net primary production, are essential for carbon flow within marine food webs and biogeochemical cycles (7). Approximately 50% of the carbon fixed by diatoms in the marine ecosystem is released into the surrounding environment, where it is subsequently absorbed and utilized by heterotrophic bacteria (8). A thin layer, known as phycosphere, surrounds the diatoms and serves as the region where molecules disperse along diffusion gradients (9). Bacteria settle in the phycosphere through random encounters, chemotactic movements, or vertical propagation (10, 11). The interactions between diatoms and bacteria typically encompass mutualism, antagonism, and parasitism (11). Extensive research has found the mutualistic or antagonistic mechanisms of bacteria against diatoms in the phycosphere (12, 13). Within the phycosphere, mutualistic bacteria, like *Ruegeria pomeroyi*, can stimulate the growth of diatom by secreting Vitamin B_12_ (VB_12_). Furthermore, they engage in a symbiotic exchange where the bacteria provide VB_12_ to diatoms and acquire organic sulfur compounds, such as 2,3-dihydroxypropane-1-sulfonic acid from diatoms (14), whereas antagonistic bacteria often inhibit the growth of phytoplankton by competition or secreting algicidal agents (12). Furthermore, it has been reported that bacteria in the phycosphere have cooperative alliances and work together to decompose and utilize algal metabolites (15, 16), suggesting a prominent role of bacterial community around the phycosphere.

However, the interaction of phytoplankton with bacteria may not always be stable. For instance, it has been reported that the release of dimethylsulfoniopropionate (DMSP) from diatoms may mediate the transition of some bacteria from mutualism to antagonism (17). Furthermore, in controlled laboratory conditions, the relationship between *Synechococcus* and heterotrophic bacteria also changes from antagonism to mutualism, ultimately evolving into symbiosis (18). To date, only one reported instance found that an elevation in cultivation temperature can alter bacteria from mutualism to antagonism with *Emiliania huxleyi* (19). Detailed reports regarding the changes in diatom-bacteria interactions under warming conditions are currently lacking. Nevertheless, elevated temperatures can exert a significant influence on diatom growth. For instance, the growth of *Skeletonema dohrnii* and *Thalassiosira pseudonana* increases with rising temperatures, while that of *S. costatum* and *Phaeodactylum tricornutum* decreases (20). For phycosphere bacteria, the increase in temperature can enhance their attachment to diatoms (11), as well as the carbon and nitrogen flux between them (21). Moreover, elevated temperature was never observed to result in a greater abundance of attached bacteria compared to free-living bacteria (21, 22). However, further assessment is still needed to evaluate how elevated temperatures induced alterations in bacterial community compositions and functional features associated with diatom.

*Skeletonema* is a ubiquitous diatom genus, which is broadly distributed from the Antarctica and the Arctic to tropical waters, and it can form large-scale blooms in coastal regions (23). To deepen our understanding of the interactions between diatom and bacteria under ocean warming conditions, we set up experiments with an axenic diatom, *S. costatum*, and investigated its interactions with bacterial community from natural seawater. We recently obtained an axenic *S. costatum* from a eutrophic bay, Xiangshan Bay (XSB). To test the hypothesis that the interactions between diatom and bacteria exhibit distinct patterns under different temperatures, natural bacteria communities from XSB and *S. costatum* were cultured together at 20°C and 25°C, enabling the investigation of the roles of algal attached and free-living bacterial community compositions and functional potentials. Moreover, *S. costatum* was also cultured with bacteria isolated from the above co-culture system at 25°C, to elucidate primary mechanisms that alter the interactions between *S. costatum* and bacteria under different temperature conditions. The results of this study will increase our understanding of how global warming alters interactions between phytoplankton and bacteria.

## MATERIALS AND METHODS

### Establishment of an axenic *S. costatum* culture

*S. costatum* was isolated in 2022 from the XSB (121°25′E–122°30′E, 29°25′N–29°47′N), China. Phytoplankton net samples were collected from the surface seawater and single cells or short chains were isolated to clonal cultures. *S. costatum* was cultured in *f*/2 + Si medium at 20°C and a 12 h light/dark diurnal cycle (70 µmol photons m^2^ s^−1^) and was rendered axenic per previously reported methods (24). Briefly, *S. costatum* was first treated with Triton-X 100 (Sigma Aldrich, Germany), and then transferred into sterile *f*/2 + Si (100 mL) augmented with antibiotics, including ampicillin (100 µg mL^−1^), gentamicin (67 µg mL^−1^), streptomycin (50 µg mL^−1^), ciprofloxacin (20 µg mL^−1^), and chloramphenicol (2.2 µg mL^−1^) and incubated for 2 days. To obtain an axenic culture, the alga was refreshed, and the above procedure was repeated two times. Then, the culture was stained with 4′,6-diamidino-2-phenylindole (DAPI, Thermo Fisher Scientific, Germany) and observed under the fluorescence microscope. Moreover, the diatom culture was introduced into 2216E medium to assess axenic status. Finally, the axenic culture was examined by scanning electron microscope (SEM, JSM-7800F, Japan). The axenic *S. costatum* was then cultured at 20°C and 25°C in a 12 h light/dark diurnal cycle (70 µmol photons m^2^ s^−1^).

### Cultivation of *S. costatum* with marine bacteria community at different temperatures

The natural seawater was collected from XSB. After filtration through a 3 µm pore size membrane, 2 L of seawater was used to culture the exponential phase growth of *S. costatum* from 20°C (NSB20) and 25°C (NSB25) to achieve a final concentration of 10,000 cells/mL for each. For the control group, natural seawater was filtrated through a 0.2 µm pore size membrane to remove all bacteria, and then the *S. costatum* was inoculated into the filtrate with the same operations described above (CK20 and CK25 for 20°C and 25°C, respectively). Finally, the co-culture systems were then incubated at 20°C and 25°C with a 12 h light/dark diurnal cycle (70 µmol photons m^2^ s^−1^). Each group had four replicates.

Samples were collected at 24, 48, 96, and 168 h. Then, 1 mL of sample was fixed with Lugol’s solution to count algal cells by the microscope (EX21; SUNNY, Ningbo, China). For bacterial community analysis, 100 mL of the control and co-culture were first filtered through 3 µm membranes to collect algal-attached bacteria (AA), and then through 0.2 µm membranes to acquire free-living bacteria (FL). The membranes were then stored at −80°C until DNA extraction. Additionally, to capture the interactions between bacteria and algal cells under different temperatures, 5 mL of co-culture was fixed with 2.5% glutaraldehyde at 4°C in the dark for 12 h, before staining with DAPI and analysis by the laser scanning confocal microscope (LSCM; LSM 880; ZEISS, Jena, Germany).

### Isolation and identification of bacteria associated with algal growth under high temperature

During the above co-culture experiments, *S. costatum* died quickly under 25°C. Then, bacteria associated with algal growth under high temperatures were also isolated. After 168 h, the co-culture (200 µL) from 25°C was inoculated into a 2216E agar plate to isolate bacteria, which were then purified. To assess the impact of bacterial isolates on algal growth, the isolated bacteria were inoculated into *S. costatum* culture at 20°C and 25°C. Finally, an isolate named MS1, exhibiting heat-induced lethal effects on *S. costatum*, was selected.

The strain MS1 was inoculated into 2216E broth and cultured at 30°C for 24 h. For bacterial identification, the genomic DNA was extracted with the help of a TlANamp Bacteria DNA Kit (TIANGEN, Beijing, China), and quantified by a NanoDrop spectrophotometer. After that, the universal primers 27F and 1492R (25) were used to amplify 16S rRNA gene. The PCR reaction was 50 µL and comprised template DNA (2 µL), Taq buffer (5 µL; Takara, Japan), forward and reverse primers (10 pmol, respectively), dNTPs (0.2 mmol/L; Takara, Japan), and Taq polymerase (0.5 U; Takara, Japan). The PCR protocol consisted of an initial denaturation step at 95°C for 5 min, followed by 35 amplification cycles with primary denaturation at 94°C for 1 min, annealing at 55°C for 1 min, extension at 72°C for 1 min. A final extension was performed for 7 min at 72°C. Furthermore, a 1,500 bp fragment was purified and utilized for sequencing by Sangon Biotech Co., Ltd. (Shanghai, China). Moreover, Mega X software (26) was employed to construct a phylogenetic tree using the maximum likelihood method with 1,000 bootstrap replicates, incorporating closely related sequences from the NCBI database. The phylogenetic tree was built according to MEGA X program (26) with a consistent bootstrap value set at 1,000.

The genome of MS1 was sequenced by Single Molecule, Real-Time (SMRT) technology at the Beijing Novogene Bioinformatics Technology Co., Ltd. SMRT Link v8.0 was utilized to filter the low-quality reads, which were then assembled using software Canu to generate one contig without a gap.

### Co-culture of *S. costatum* and MS1

The MS1 (1 × 10^6^ cells/mL) in exponential phase was added in the culture (40 mL) of exponentially growing *S. costatum* (1 × 10^4^ cells/mL) from 20°C and 25°C. For the control group, an equal volume of sterile 2216E broth was added to the *S. costatum* culture. The co-culture systems were then incubated at 20°C and 25°C and 12 h light/dark diurnal cycle (70 µmol photons m^2^ s^−1^). Each group had three triplicates.

Sampling was performed at 12, 24, 48, 72, and 120 h. The relative fluorescence units (RFUs) of *S. costatum* were measured with an excitation and emission wavelength of 440 and 680 nm, respectively (27). LSCM analysis was conducted as described above. The interaction between *S. costatum* and MS1 was further observed using field emission SEM by following the sample preparation protocol as reported previously (28). Briefly, 1 mL of the samples was collected and washed three times with sterile *f*/2 broth. Then, 20 µL of this washed sample was evenly coated on a 50 mm × 50 mm silicon wafer, air-dried in a clean bench, fixed in a 2.5% glutaraldehyde solution, and then stored at 4°C for 12 h. Then, the samples were subjected to ethanol dehydration in ascending concentrations of 30%, 50%, 70%, 90%, and 100%. Following air-drying, the samples were frozen at −80°C for 2 h, freeze-dried, and then coated with metal before observing cell morphology.

### Bacterial 16S rRNA gene amplicon sequencing and data processing

A total of 68 DNA samples were extracted using the Power Soil DNA Isolation Kit (MOBIO, USA). The 16S rRNA gene’s V4-V5 region was amplified with primers 515F-Y (5′-GTGYCAGCMGCCGCGGTAA-3′) and 926R-B (5′-CCGYCAATTYMTTTRAGTTT-3′) (29). For library sequencing, an Illumina MiSeq platform (Illumina, San Diego, CA, USA) was used, which generated 2 × 250 bp paired-end reads. The detailed method of library construction is present in our previous study (30).

The QIIME2 pipeline was used for processing paired-end reads (31). Briefly, raw reads were trimmed and denoised using DADA2 (32) to generate amplicon sequence variants (ASVs). For taxonomic assignments, each representative sequences were aligned against the SILVA 138 database (33). ASVs, identified as archaea, chloroplast, mitochondria, and unassigned ASVs were discarded. Finally, an ASV feature table comprising 9,313 ASVs was acquired, and samples were further rarefied to a sequence depth of 22,300 for the following analyses.

### Prediction of functional potentials of bacterial community

The PICRUSt2 pipeline was employed to infer the functional potentials of the bacterial community (34). Furthermore, a table of Kyoto Encyclopedia of Genes and Genomes (KEGG) Orthologs (KOs) was generated by PICRUSt2, based on the ASV feature table. Then KEGG Mapper was employed to reconstruct tables of KEGG reference categories (KEGG level 1) and modules (KEGG level 2) based on the KO annotations (35). Moreover, KOs related to motility and chemotaxis, carbon and nitrogen metabolism, as well as DMSP metabolism of AA and FL were selected. Finally, the differences between these KOs at 20°C and 25°C were tested by stamp software (36).

### Statistical analysis

Using the Linear Discriminant Analysis (LDA) Effect Size (LEfSe) method (37), differentially abundant taxa within AA and FL at different temperatures were identified. Non-metric multidimensional scaling (NMDS) ordination, relying on Bray-Curtis dissimilarity, was employed to visualize variations in the bacterial community structure. Furthermore, the significance of stochastic processes was assessed by determining the stochasticity ratio (ST), which was evaluated by comparing the ratio of the mean expected similarity of the observed similarity to the null communities (38). Null communities were generated by a null model algorithm comprising 1000 randomizations of the observed community. Moreover, the tNST function in the “nst” package in R was employed for assessing the ST ratio (38). The ST value >0.5 indicated stochastic processes, while an ST value <0.5 indicated deterministic processes.

For the co-occurrence network at 20°C and 25°C, Spearman’s rank correlations among ASVs were assessed, and only strong (|*ρ*| >0.6) and significant (FDR-adjusted *P* < 0.01) correlations were kept for network analysis by using “igraph” package in R (39). Finally, Gephi was used for co-occurrence network generation (40).

### Genome comparison

The MS1 genome annotation was conducted using Prokka (41). Furthermore, comparative genomic analysis was performed using the Proksee tool (https://proksee.ca/) to generate circular views of the complete genomes of MS1 and *Tamlana crocina* HST1-43 (GCF_012037625.1). Moreover, Pyani was employed for calculating the average nucleotide identities (ANI) (42). Genome-to-genome Distance Calculator 3.0 (GGDC) was used to analyze digital DNA-DNA hybridization (dDDH) between bacteria (43). The dDDH comparisons were performed using GGDC between the genome of MS1 and genomes from the *Tamlana* genus (43), with a threshold of 70%. Additionally, a pan-genome analysis was also performed by the Bacterial Pan Genome Analysis (BPGA) Tool v1.3 (44), wherein for clustering USEARCH was employed with a sequence identity cutoff of 50%. The genomic annotation was conducted by referring to the KEGG and Clusters of Orthologous Groups of proteins (COG) database. Carbohydrate-active enzymes (CAZymes) were identified based on HMMER searches (HMMer 3.0b) (45) against the dbCAN2 (46).

## RESULTS

### Succession of the bacterial community associated with *S. costatum* under different temperature

**Fig 1:**
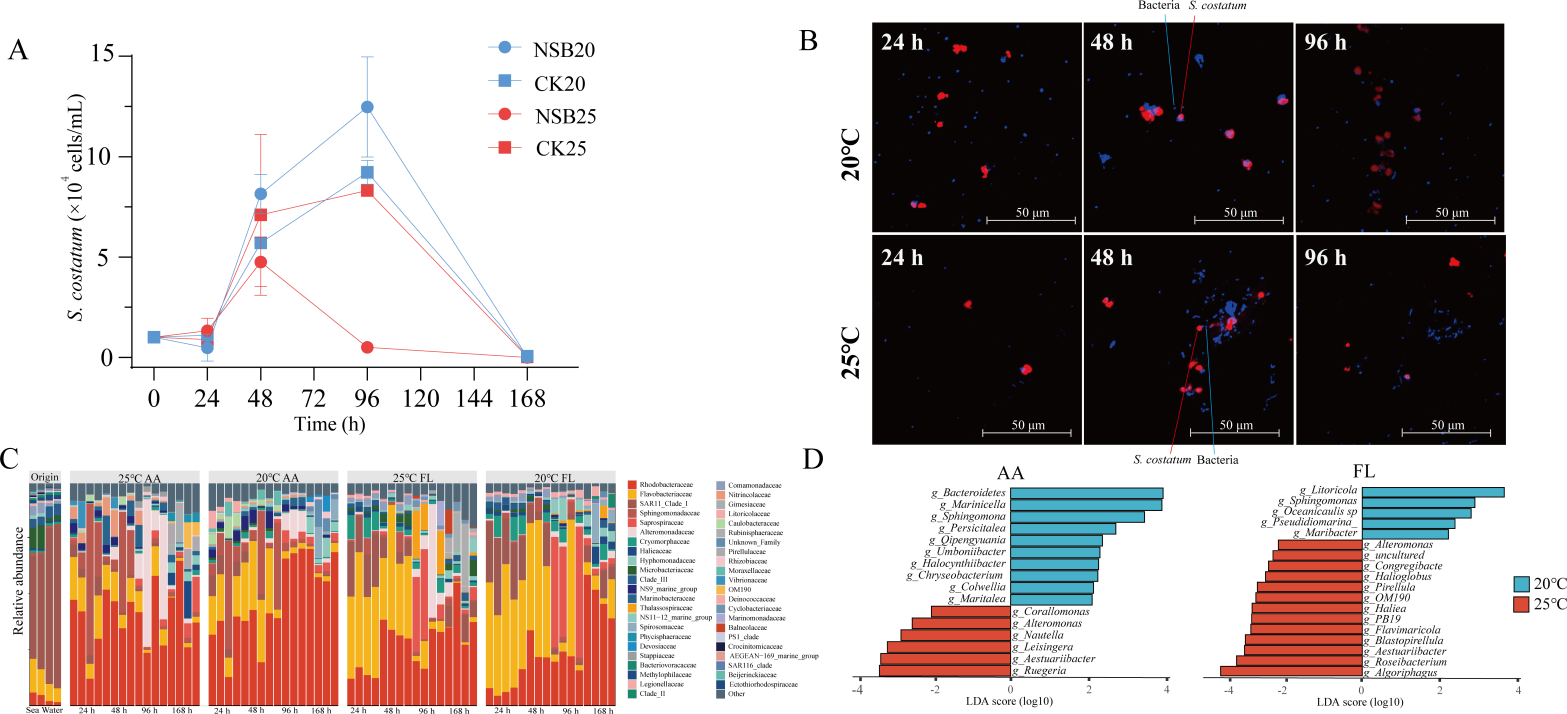
Relationship between *S. costatum* and bacterial community in the co-culture system under different temperature conditions. (A) The growth of *S. costatum* with natural seawater bacteria community at 20°C and 25°C. CK20 and CK25 indicate the control group at 20°C and 25°C, respectively. NSB20 and NSB25 indicate the alga co-cultured with natural seawater bacteria at 20°C and 25°C, respectively. (B) Interactions between *S. costatum* and bacteria observed by laser scanning confocal microscopy. Bacteria and *S. costatum* were colored by blue and red, respectively. (C) Changes of bacterial community compositions in the co-culture system. Bacteria with average relative abundances >2% were selected. (D) Linear discriminant analysis of differential bacteria in two groups. AA, algal-attached bacteria; FL, free-living bacteria.

In the control group, *S. costatum* exhibited a similar growth pattern at both 20°C and 25°C (Fig. 1A), undergoing an exponential growth phase, reaching its peak abundance at 96 h, and subsequently experiencing mortality with a notable rise in mortality rate. For the NSB20 group, the growth pattern closely resembled that of the control group. However, at 25°C, *S. costatum* underwent lysis at 48 h with its mortality rate reaching 94.3% by 96 h (Fig. 1A). In all groups, *S. costatum* underwent complete lysis by 168 h, possibly associated with the oligotrophic nutrients of the natural seawater. The LSCM observation found that at 48 h, the number of bacteria surrounding *S. costatum* cells in NSB25 was markedly higher than that observed in NSB20 (Fig. 1B). Furthermore, there was a noticeable decrease in the presence of intact *S. costatum* in NSB25 at 96 h, with bacteria predominantly residing around the debris of algal cells.

In origin seawater, SAR11_Clade I was the dominant bacteria (Fig. 1C). However, after co-culture with *S. costatum*, *Rhodobacteraceae* and *Flavobacteriaceae* were the dominant bacteria in NSB20 and NSB25. Furthermore, within 48 h, the relative abundance of *Flavobacteriaceae* was remarkably higher in FL than in AA; however, after 48 h, its abundance decreased significantly in both AA and FL communities, suggesting a close relationship with the dynamics of *S. costatum*. Compared to the AA community in NSB25, the relative abundances of *Rhodobacteraceae* were significantly higher in the NSB20 of the AA community after 96 h. Conversely, the AA community in the NSB25 showed an increased abundance of *Saprospiraceae*, *Alteromonadaceae*, *Halieaceae*, and *Gimesiaceae* (Fig. 1C).

The LDA analysis unveiled six discriminant genera within the AA community at 25°C, all of which belonged to *Rhodobacteraceae*. Among them, *Ruegeria* had the highest LDA score, followed by *Aestuariibacter*, *Leisingera*, and *Nautella* (Fig. 1D). Conversely, at 25°C, the discriminant genera for FL community were more diverse than those of AA community, including *Algoriphagus*, *Roseibacterium*, *Aestuariibacter*, *Blastopirellula*, and so on (Fig. 1D).

### Effects of elevated temperature on the relationships between bacterial community and *S. costatum*

**Fig 2:**
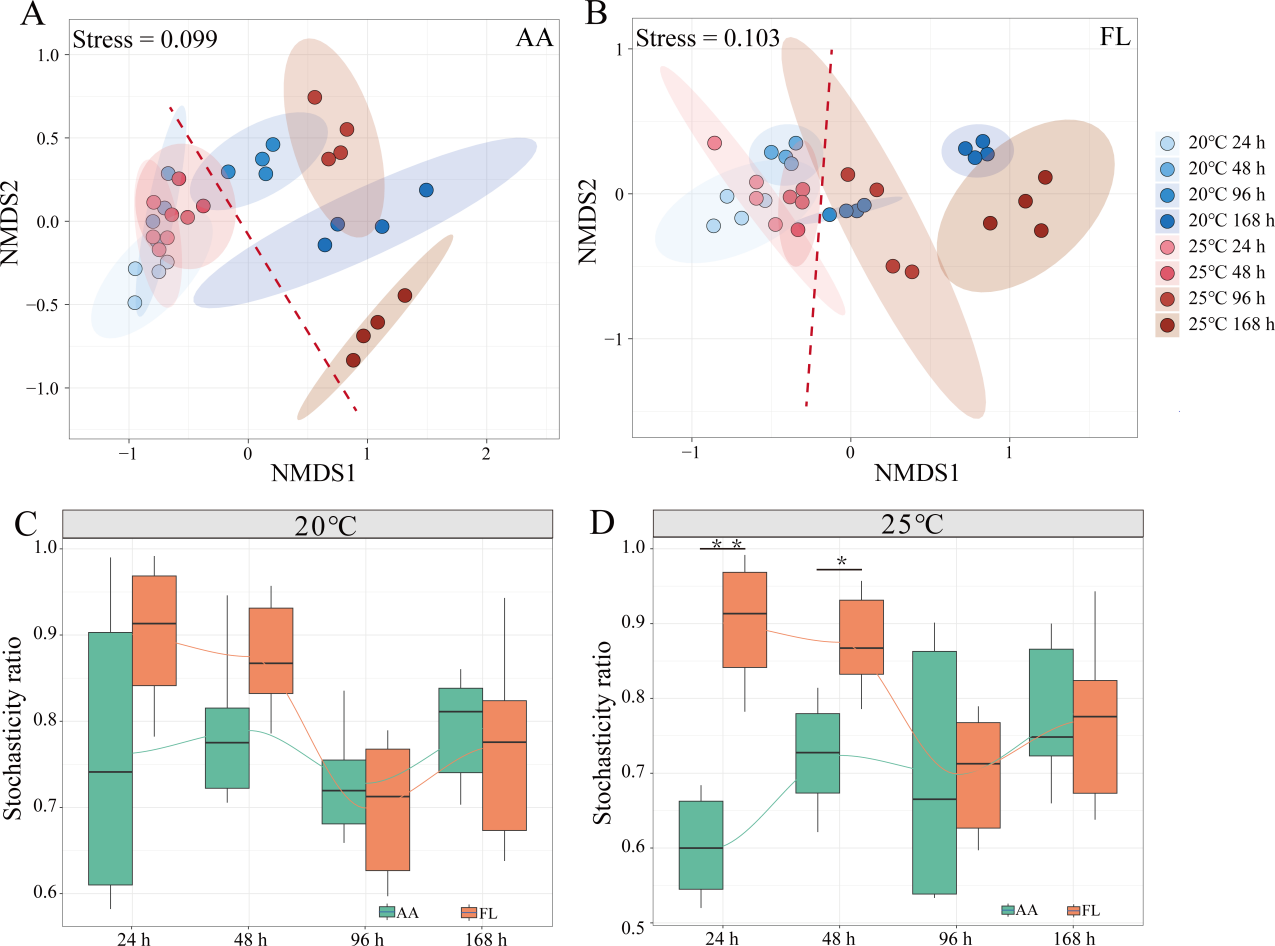
Changes of bacterial community in the co-culture system. (A and B) Non-metric multidimensional scaling (NMDS) plots of the algal-attached (AA) and free-living (FL) bacterial community at 20°C and 25°C, respectively. The red dashed line separated bacterial community before and after 96 h, when the algae lysed. (C and D) Stochasticity ratios (SRs) of bacterial community at 20°C and 25°C, respectively. An SR value greater than 0.5 indicates stochastic processes, while an SR value less than 0.5 indicates deterministic processes. **P < 0.01, *P < 0.05.

According to the NMDS analysis, the successional patterns in the AA and FL communities were similar (Fig. 2A and B). Briefly, both bacterial communities moved along with the NMDS1 axis. In the AA community, the NSB20 and NSB25 samples were closely clustered before 48 h; however, they showed a more distinct grouping based on temperature and co-culture time in the FL community. After 96 h, both AA and FL communities clustered separately based on the temperature and sampling time. Moreover, both AA and FL communities indicated significant differences before and after the death of *S. costatum*. The assembly of bacterial communities was driven by stochastic processes, with the ST values in the system consistently exceeding 0.5 (Fig. 2C and D). In the initial 48 h, while the algal cells were alive, the ST values of the AA community were less than those of the FL community. However, after 96 h, the stochasticity of both communities was comparable in NSB20 and NSB25. The trends in ST at higher temperatures were more significantly associated with the dynamics of *S. costatum*.

**Fig 3:**
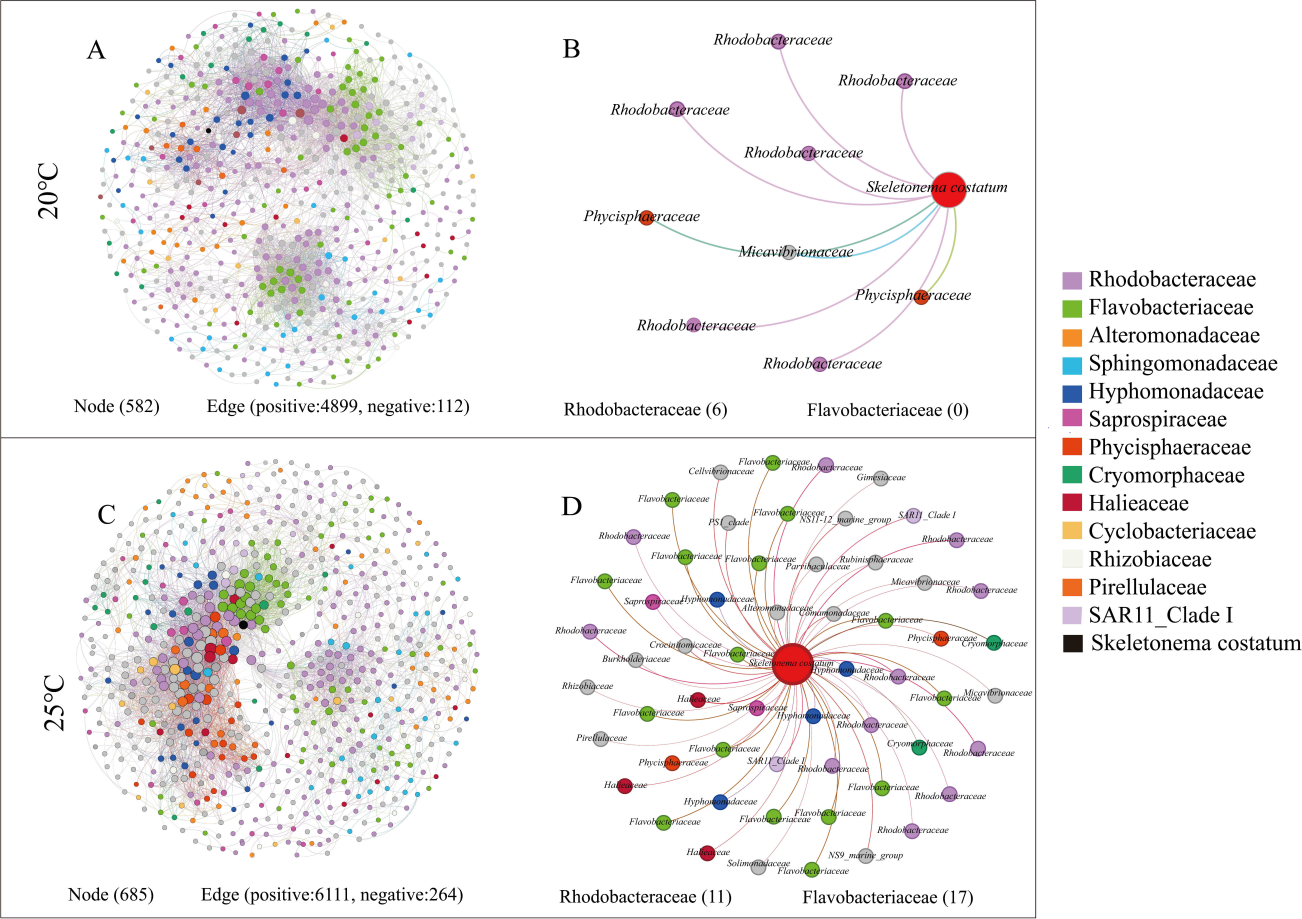
Co-occurrence networks between *S. costatum* and bacteria community. (A and C) Network analysis revealed co-occurrence patterns between *S. costatum* and bacteria at 20°C and 25°C, respectively. (B and D) Bacteria exclusively associated with *S. costatum* in the network at 20°C and 25°C, respectively.

Co-occurrence network analysis elucidated a significant increase in the complexity of interactions after the increase in the temperature (Fig. 3A and C). The number of nodes in the network increased from 582 at 20°C to 685 at 25°C, with increasing edges from 5,011 to 6,375. At 20°C and 25°C, the positive correlations among nodes dominated the network edges. Furthermore, the interactions of *S. costatum* with bacteria were substantially increased from 20°C to 25°C, with bacterial nodes increasing from 9 to 58. At 20°C, six out of nine bacterial nodes were predominantly affiliated with *Rhodobacteraceae* (Fig. 3B). In contrast, at 25°C, the bacterial nodes coexisting with *S. costatum* were primarily dominated by *Flavobacteriaceae* (17), followed by *Rhodobacteraceae* (11) (Fig. 3D). As inferred, the increase in temperature enhanced the correlations between *Flavobacteriaceae* and *S. costatum*.

### Response of functional potential to elevated temperature

**Fig 4:**
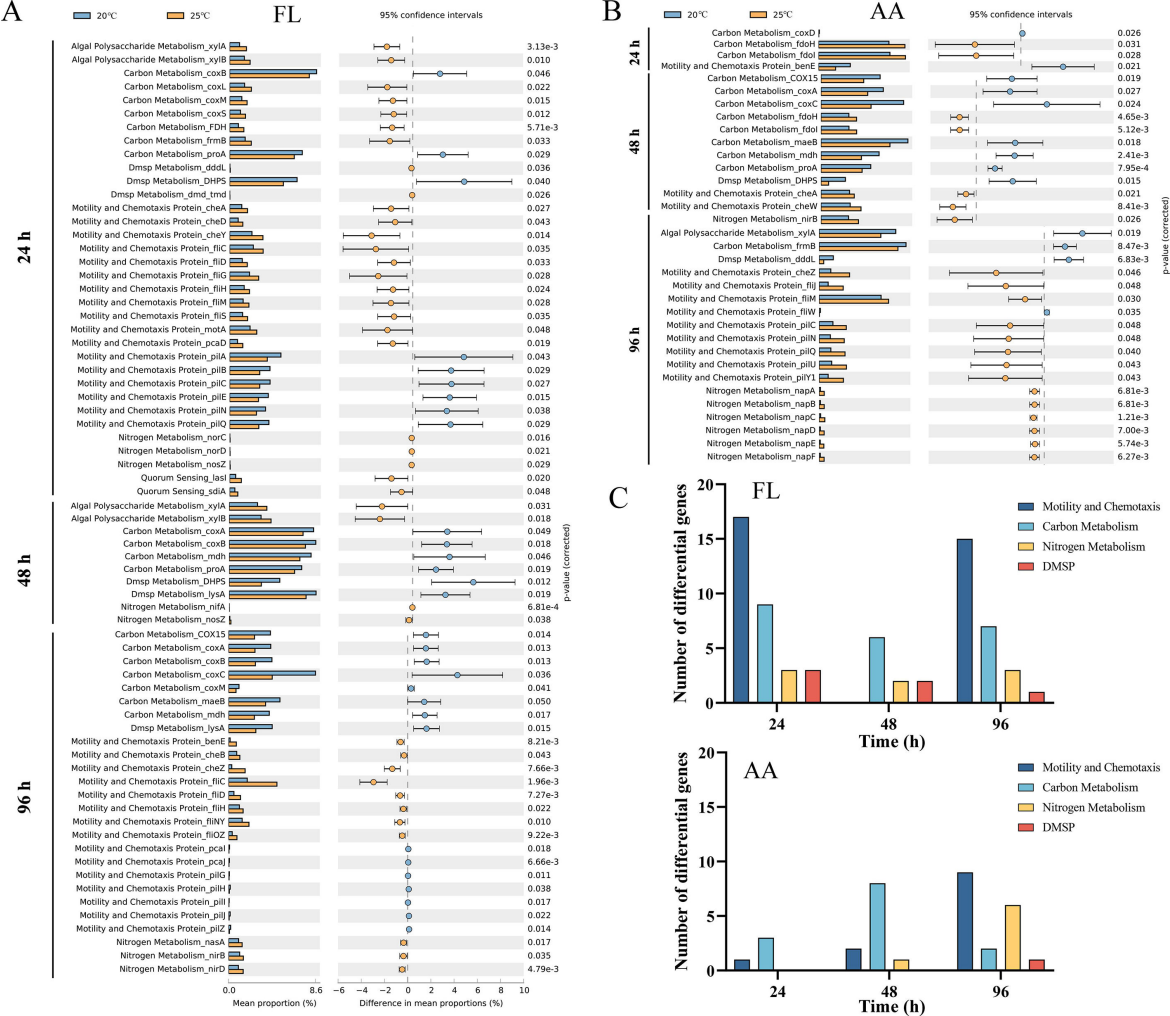
PICRUSt2 predicts the functional potential of bacterial community in the co-culture system. (A and B) Functional potential of free-living (FL) and alga-attached (AA) bacteria, respectively. (C) Number of predicted genes in the four metabolic pathways of free-living (top) and alga-attached (bottom) bacteria.

The impact of elevated temperature on the functional potential of FL community was markedly more pronounced than the AA community (Fig. 4A and B). Furthermore, in the FL community, the relative abundance of 68 genes exhibited significant variations across different temperatures (Fig. 4C). However, the AA community displayed a more constrained response, with only 33 genes showing variations within these specific functional categories (Fig. 4A and B). In the FL community, the majority of differentially abundant genes, notably 17 associated with motility and 15 with chemotaxis, were observed at both 24 and 96 h, whereas no significant differences in the abundance of motility and chemotaxis genes were noted at 48 h (Fig. 4A). Additionally, for motility and chemotaxis, the type IV pili genes, including *pilA*, *pilB*, *pilC*, *pilE*, *pilN*, and *pilQ*, were significantly enriched in NSB20 compared with NSB25. In contrast, the flagellar-related genes, including *fliC*, *fliD*, *fliG*, *fliM*, *fliH*, and *fliS*, were significantly enriched in NSB25 than in the NSB20 group. Furthermore, it was observed that at 96 h, eight out of nine motility and chemotaxis genes, including type IV pili and flagellar-relate genes were enriched in NSB25 of the AA community. Moreover, the nitrogen metabolism-related genes, mainly nitrate reductase were more abundant in NSB25 of the AA community (Fig. 4A and B).

### Isolation of MS1 that changes from mutualism at 20°C to antagonism at 25°C

**Fig 5:**
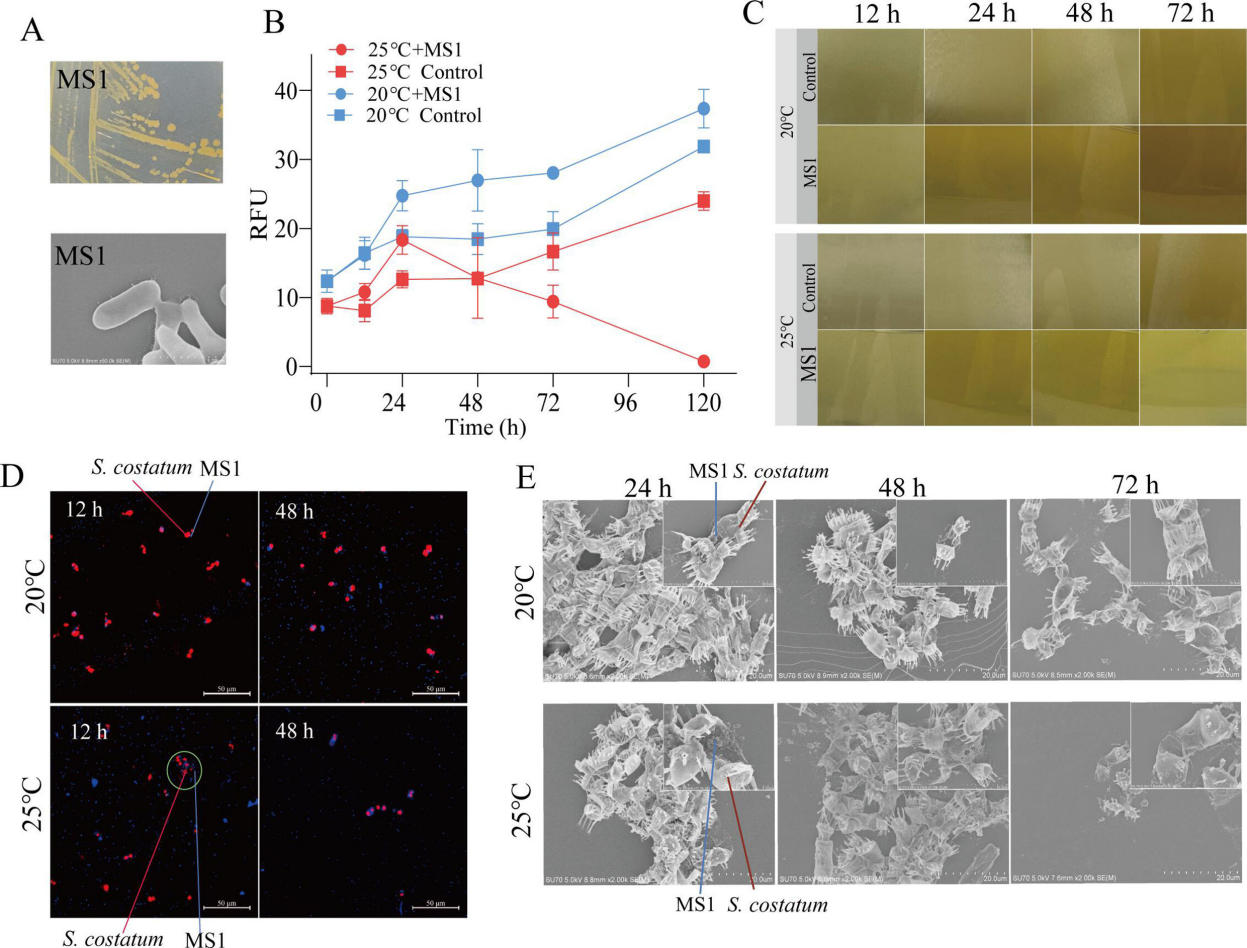
Interactions between MS1 and *S. costatum*. (A) Morphology of MS1. (B) Effects of MS1 on the growth of *S. costatum* under different temperatures. (C) Changes in the appearance of the co-culture system. (D and E) The attachment of MS1 to *S. costatum* cells observed by laser scanning confocal microscopy (D) and scanning electron microscopy (E).

From the NSB25 group, 56 bacterial strains were isolated. After co-culturing with *S. costatum* at 20°C and 25°C, a yellow, short rod-shaped bacterium (MS1) was identified to have the most pronounced temperature-induced lethal effects (Fig. 5A). The RFU of *S. costatum* increased rapidly in the control group at both 20°C and 25°C; however, it was slightly higher at 20°C than at 25°C (Fig. 5B). When co-cultured with MS1 at 20°C, the RFU of *S. costatum* increased more than that observed at 20°C (Fig. 5B). Furthermore, at 25°C, MS1 exhibited a significant ability to kill *S. costatum* after 24 h, with the mortality rate gradually increasing from 30.2% at 48 h to 92.4% at 120 h (Fig. 5B). The transition in the lifestyle of MS1, from mutualism at 20°C to antagonism at 25°C, was also evident from the color change of the co-culture system (Fig. 5C). The color shifted from a brownish-yellow to a light yellow-transparent color, whereas at 20°C, the color gradually darkened (Fig. 5C). The LSCM observed that MS1 attaches to *S. costatum* cells at both 20°C and 25°C, with substantial accumulation of MS1 observed in aggregation regions of *S. costatum* cells (Fig. 5D). Additionally, within the initial 24 h, there was increased number of MS1 cells attached to algal aggregates at 25°C compared to 20°C (Fig. 5E). At 25°C, a higher incidence of broken algal cells was observed, and MS1 accumulated more prominently on these damaged cells (Fig. 5E). Overall, these data indicated that elevated temperature alters MS1 lifestyle from mutualism to antagonism.

### MS1 is a potential novel *Tamlana* species closely related to *Tamlana crocina*

**Fig 6:**
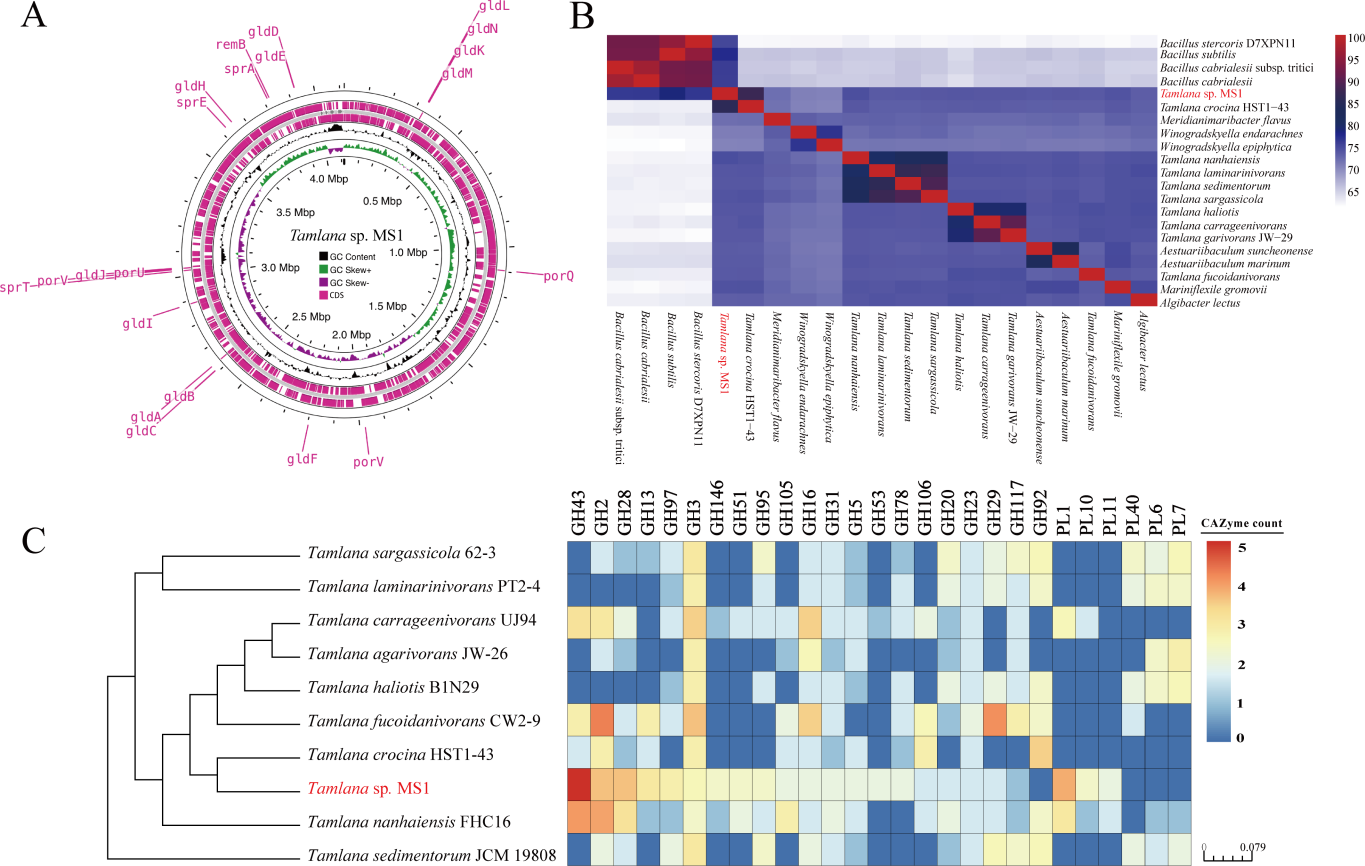
Genomic information of MS1. (A) Gene location of gliding motility and T9SS. (B) Average nucleotide identity analysis for MS1. (C) Number of genes in each CAZyme family (right), clustered by a maximum-likelihood phylogenetic tree based on core genes (left).

MS1 was identified as the genus of *Tamlana* within *Flavobacteriaceae* family based on the 16S rRNA gene sequence (Fig. S1). Furthermore, MS1 had the highest similarity with *Tamlana crocina* HST1-43 (98.7% similarity), followed by *Aestuariibaculum suncheonense* (94.8% similarity). The complete genome sequence of MS1 was 4.12 Mb, containing 3,467 genes (Fig. 6A; Table S1). The dDDH value between MS1 and *T. crocina* HST1-43 was 19.4%, which was the highest among the bacteria compared (Table S2). This value fell below the accepted species threshold of 70%. The ANI analysis further demonstrated that MS1 shares the highest similarity (85.5%) with *T. crocina* HST1-43 (Fig. 6B), a value clearly below the commonly accepted species demarcation threshold of 95%. Altogether, these results suggested that strain MS1 may represent a novel species within the *Tamlana* genus.

Interestingly, the genome of MS1 conspicuously harbors 20 genes related to gliding motility and the type IX secretion system (T9SS), including *gldA-F*/*H-N*, *sprA/E/T*, and *porV/U/Q* (Fig. 6A). The KEGG analysis indicated that genes were mainly enriched in metabolic pathways (452), secondary metabolites biosynthesis (215), antibiotics biosynthesis (164), and microbial metabolism in diverse environments (136) (Fig. S2A). Furthermore, 2,422 proteins were identified by COG database, of which the most abundant genes were involved in carbohydrate transport and metabolism (251) and cell wall/membrane/envelope biogenesis (249), followed by general function (209) and amino acid transport and metabolism (200) (Fig. S2B).

### Comparative genomics analysis of *Tamlana* sp. MS1

Comparative genomics showed that the number of core genes for the 10 *Tamlana* genus is 1,464, and MS1 has 788 special genes (Fig. S3A). For CAZymes, MS1 had the highest abundance of CAZymes, including 166 glycoside hydrolases (GH) and 25 polysaccharide lyases (PL) (Fig. S3B). Among these enzymes, GH43 (32) and PL1 (13) indicated the highest expression than other strains (Fig. 6C). The genomic functional predictions of strain MS1 indicate proficient capabilities in vitamin synthesis and transport, such as the capacity to synthesize biotin, folate, thiamine, Vitamin K_1_, and transport VB_12‍_; however, MS1 cannot synthesize VB_12_. MS1 genome encodes various predicted pathways for utilizing diverse organic and inorganic compounds, including those known to exist in diatoms (Table S3). Moreover, MS1 also predictably encoded polyphosphate kinase enzymes that can degrade and utilize diatoms generated phosphorous source polyphosphate. Overall, these genes may facilitate the observed physical interaction with diatom cells.

## DISCUSSION

In marine ecosystems, phytoplankton and their associated bacterial community collectively participate in several key biological processes. In the phycosphere, heterotrophic bacteria employ diverse mechanisms to establish symbiotic relationships with phytoplankton cells and engage in metabolite exchange. Temperature could significantly alter the metabolic characteristics of phytoplankton, consequently influencing bacterial community compositions in the psychosphere (47). Our findings revealed that during increased temperatures, the natural seawater bacterial community exerts an inhibitory effect on the growth of *S. costatum*. Furthermore, these elevated temperatures can stimulate the growth of bacteria as well as *S. costatum*, hastening nutrient depletion and ultimately leading to algal collapse (48). However, this phenomenon was not observed in the present study, as the growth of *S. costatum* was inhibited after only 48 h. Therefore, it was speculated that the death of algae might be linked with high temperatures-induced lifestyle alterations of bacteria.

*Rhodobacteraceae* and *Flavobacteriaceae* were observed as dominant bacteria in the co-culture system at both 20°C and 25°C, consistent with previous studies on algal-associated bacterial communities (49, 50). Members of *Rhodobacteraceae* can secrete VB_12‍_, which supports the growth of diatoms (51). Therefore, *Rhodobacteraceae* can rapidly establish symbiotic relationships with diatoms. Because of their abilities to utilize algal polysaccharides, members of the *Flavobacteriaceae* frequently occupy advantageous positions in algal-associated environments (52, 53). However, after the algal cell lysis, a notable decrease was observed in the relative abundance of *Flavobacteriaceae*, indicating a close association with the growth of *S. costatum*. This phenomenon is frequently observed during algal bloom, where *Flavobacteriaceae* thrive amidst extensive phytoplankton proliferation, displaying a preference for high-molecular-weight dissolved organic matter (DOM) (54). Although *Rhodobacteraceae* employ various survival strategies, from streamlined oligotrophs to metabolically versatile opportunists. They can degrade phytoplankton-derived low-molecular-weight DOM when interacting with phytoplankton (55). This effectively clarifies why the abundance of *Rhodobacteraceae* was markedly higher in AA than the FL. Furthermore, due to their diverse survival strategies, *Rhodobacteraceae* can still maintain high abundance after the lysis of the *S. costatum*. Additionally, LDA analysis revealed prominent discriminative bacteria from *Rhodobacteraceae* in the AA at 25°C. High temperature could improve the demand of diatom for VB_12 ‍_ (56), thereby favoring *Rhodobacteraceae* selection in the phycosphere. The discriminative bacteria, including *Rueger* and *Nautella*, have been identified as VB_12‍_ producers (57), which were selected by *S. costatum*. Moreover, bacterial communities in both AA and FL demonstrated a significant connection with the growth of *S. costatum*, as evidenced by NMDS analysis, indicating distinct divergence in bacterial community structures upon algal demise. This further highlighted that DOM from *S. costatum* shapes both AA and FL bacterial communities.

Elevated temperature increases the intensity of interactions among bacteria, as well as between bacteria and *S. costatum*. Temperature cannot only increase the release of DOM by diatoms (58) but also enhance the attachment of heterotrophic bacteria to algal cells, promoting carbon-nitrogen flux (21, 59). This was confirmed by LSCM, which found intensive attachment of bacteria to *S. costatum* at 25°C (Fig. 1B). Interestingly, no interactions between *Flavobacteriaceae* and algae were observed at 20°C, yet their interactions increased remarkably at 25°C, surpassing those between *Rhodobacteraceae* and algae. This indicated that the interaction between *Flavobacteriaceae* and algae was highly sensitive to elevated temperature than those with *Rhodobacteraceae. Flavobacteriaceae* adeptly utilize new DOM released by diatoms under warming conditions (22). It was also speculated that the decline of *S. costatum* at high temperatures may be closely related to *Flavobacteriaceae* given its increased interactions with *S. costatum*. Members of *Flavobacteriaceae* are commonly reported as algicidal bacteria capable of lysing multiple algal species (60, 61), including *S. costatum*. At high temperatures, bacteria belonging to *Flavobacteriaceae* may alter their lifestyle to become more lethal to *S. costatum*.

The ST ratios are widely employed to assess the impact of ecological stochasticity on microbial community assembly (38). Here, stochastic processes predominantly modulated the process of entire community assembly; however, it was notably decreased in stochasticity at the time of *S. costatum* mortality (Fig. 2C and D). By combining redundancy (62) and the lottery hypotheses (63), within groups of species sharing similar ecological characteristics, the first arrival secures the “lottery” of niche. However, it was also found that bacterial community associated with *Thalassiosira rotula* was driven more by deterministic processes because the ecological niche surrounding diatoms can offer bacteria species-specific metabolic features, leading to a highly stable and repeatable core bacterial community (49). The results of the present study more evidently align with the former perspective. However, stochasticity was lower in the AA than in the FL community, and this difference became more pronounced under elevated temperatures. This suggests the constraining effect of phycosphere around diatoms on community assembly, which was amplified under high temperatures. Moreover, with the mortality of *S. costatum*, a convergence effect occurred on community assembly, delineating bacterial community structures into two phases attributed to the death of *S. costatum* (Fig. 2A and B). The released DOM from *S. costatum* can strongly alter the nutrient levels in the co-culture system, thus decreasing the stochasticity of the bacterial community. Furthermore, at high temperatures, the relatively high stochasticity in FL was also linked with the motility of bacteria. FL community was more significantly impacted by higher temperatures, resulting in significant differences in the relative abundance of a large number of genes, particularly those related to motility and chemotaxis. At high temperatures, the FL community motility shifted from being dominated by pili to being governed by flagella, thus exhibiting enhanced motility (Fig. 4). Regarding diatom proliferation and algal bloom collapse, increased motility bacteria have advantages over non-motile ones to acquire more DOM (64). Additionally, the co-culture system exhibited relatively higher instability during the early (24 h) and decline stages (96 h) of *S. costatum* compared to the stability observed at 48 h, consequently resulting in more variations in gene abundance at 24 and 96 h. Moreover, the alteration of DOM may affect the behavior in the FL community. Due to the relatively stable environment in the phycosphere, the AA community had few differential motility genes at 24 and 48 h. Altogether these data revealed that alterations in stochasticity in this co-culture system were closely linked with *S. costatum* growth and bacterial motility.

*Tamlana* sp. MS1, isolated from the co-culture system at 25°C, exhibited high algicidal effects on *S. costatum* under elevated temperatures (Fig. 5), further indicating the essential activity of *Flavobacteriaceae* interacting with *S. costatum*. Research has indicated that *Ruegeria* sp. R11 displays temperature-enhanced virulence against *E. huxleyi*; however, the underlying mechanisms remain undetermined (19). Furthermore, the lifestyle of *Sulfitobacter* D7 switches from coexistence to pathogenicity after it interacts with *E. huxleyi* (65), and this mode is known as the “Jekyll-and-Hyde” phenotype. Algal DMSP has been identified as a key chemical component that mediates the transition between lifestyles (17). In its pathogenic phase, the flagellar motility and various transport systems of *Sulfitobacter* D7 were significantly enhanced, likely aiming to maximize assimilation of metabolites originating from algae following cell death. Moreover, a similar phenomenon has also been identified in the *Phaeobacter* genus when it is co-cultured with *E. huxleyi* (66, 67). However, these transitions were mainly triggered by algal senescence, which is different from the findings of this research, where transitions are induced by temperature.

When MS1 was co-cultured with *S. costatum*, a considerable amount of MS1 adhered to the surface of algal cells at 25°C, as evidenced by SEM revealing substantial aggregation of MS1 at damaged sites of algal cells. Conversely, only a small quantity of MS1 was observed on algal cells in the co-culture system at 20°C. This suggests that an elevation in temperature augments the motility of MS1, thereby facilitating its attachment to the surface of algal cells. Numerous investigations have already substantiated the intimate association between bacterial virulence and their motility and attachment (68, 69). Studies on coral pathogenic bacteria have elucidated the pivotal role of motility in positioning and initiating infection within the host during the initial stages of *Vibrio* infection, with heightened motility at elevated temperatures correlating with increased infectivity (68). Conversely, mutants with impaired motility display diminished infectivity, while non-motile mutants fail to infect corals (68). Thus, bacterial motility and attachment are intricately linked to their pathogenicity and are significantly influenced by temperature.

The gliding motility exhibited by *Flavobacteria* serves as a paradigm for studying bacterial gliding, with genes associated with gliding being widely dispersed throughout the *Flavobacteria* (70). The gliding motility system encompasses two systems: (i) the motility apparatus composed of membrane Gld subunits B, D, H, and J; (ii) the T9SS constituted by GldK/L/M/N and SprA, SprE, PorV, and others (70–72). These two systems collaboratively orchestrate the transition from bacterial motility to attachment. The T9SS demonstrates remarkable efficacy in secreting CAZymes and various extracellular proteins, while also playing a role in facilitating surface-associated gliding motility (73). Numerous investigations found the indispensability of gliding motility and T9SS in attachment, virulence, and extracellular protease secretion in *Flavobacteria* (71, 73, 74). In pathogenic species such as *Flavobacterium psychrophilum* and *F. columnare*, mutants deficient in *gldD*, *gldN*, and *porV* not only lack attachment capabilities but also exhibit markedly reduced protease activity and pathogenicity (73). Comparative genomic analysis has unveiled the presence of a complete set of *gld* and *spr* genes encoding gliding motility and T9SS in the MS1 genome, indicative of the possession of a comprehensive gliding motility and T9SS system by MS1. Furthermore, the filtrate from MS1 fermentation did not notably affect algal growth, implying that MS1-induced algal death requires direct attachment to the surface of algal cells. Consequently, we propose that elevated temperatures can potentially enhance the gliding motility of MS1, promoting its attachment to algal cells, and ultimately resulting in the death of *S. costatum*.

In general, bacteria induce algal death through two primary modes: indirect or direct (or a combination thereof, depending on the host) (75). In the indirect mode, bacteria release algicidal compounds that result in algal death, with documented instances of algicidal microbes predominantly employing this mechanism (75–77). Direct mode entails bacterial contact and attachment for effective algal demise, as exemplified by *Streptomyces globisporus* encircling *Microcystis aeruginosa* cells, leading to direct algal death (78). The marine bacterium *Saprospira* sp. SS98-5 can directly lyse *Chaetoceros ceratosporum* by utilizing gliding motility to approach diatoms, thereby inducing diatom aggregation and subsequently rupturing diatom cells through the production of microtubule-like structures (79). It is apparent that bacteria possessing gliding motility, such as MS1, have the potential to induce algal death through direct contact.

### Conclusions

*S. costatum* often forms blooms in coastal waters, significantly affecting marine ecosystems. The elevated surface seawater temperature could affect the dynamics of *S. costatum* and its interactions with bacteria. The findings of the present study indicated that natural seawater bacteria can accelerate the mortality of *S. costatum* under high-temperature conditions. The LDA and co-occurrence network analyses validated that members of *Flavobacteriaceae* promote algal mortality under elevated temperatures. Then, we demonstrated that elevated temperature could activate the “Jekyll-and-Hyde” mode of a *Flavobacteriaceae* isolate, MS1, making its lifestyle transition from mutualism to antagonism with *S. costatum*. This transition may be attributed to the increased gliding motility and attachment of MS1 under elevated temperature, enabling it to exert an algicidal effect through direct contact. Therefore, with rapid ocean warming, the phycosphere bacteria may undergo a temperature-induced lifestyle transition, thereby significantly influencing the phytoplankton biomass. This study provides new insights into phytoplankton-bacteria interaction during temperature elevation, which will increase the reference and knowledge in this field.

## Data Availability

The raw data of 16S rRNA gene and MS1 genomic sequence are deposited in the National Genomics Data Center (https://bigd.big.ac.cn/gsa), Beijing Institute of Genomics (China National Center for Bioinformation), Chinese Academy of Sciences, under accession numbers CRA014975 and CRA015017, respectively.

## SUPPLEMENTAL MATERIAL

Supplemental material msphere.00198-24-s0001.docx Tables S1 to S3; Figures S1 to S3.

ASM does not own the copyrights to Supplemental Material that may be linked to, or accessed through, an article. The authors have granted ASM a non-exclusive, world-wide license to publish the Supplemental Material files. Please contact the corresponding author directly for reuse.
