# Supplementary material for: Elevated temperature alters bacterial community from mutualism to antagonism with Skeletonema costatum: insights into the role of a novel species, Tamlana sp. MS1
Source: mSphere. 2024 Jun 28;9(7):e00198-24. doi: 10.1128/msphere.00198-24 (PMC11288006; doi:10.1128/msphere.00198-24)
Supplement: Supplemental material — Tables S1 to S3; Figures S1 to S3. [file msphere.00198-24-s0001.docx]

**Elevated temperature alters bacterial community from mutualism to antagonism with *Skeletonema costatum*: Insights into the role of a novel species, *Tamlana* sp. MS1**

Tenghui Lin ^a,c^, Yumeng Feng ^c^, Wenfei Miao ^c^, Shuqi Wang ^c^, Zhen Bao ^c^, Zeyuan Shao ^c^, Demin Zhang ^a,c,d^, Xinwei Wang ^c,d^, Haibo Jiang ^c,d^, Huajun Zhang ^a,b,c,d*^

^a^ State Key Laboratory for Managing Biotic and Chemical Threats to the Quality and Safety of Agro-products, Ningbo University, Ningbo, 315211, China

^b^ Key Laboratory of Marine Ecosystem Dynamics, Second Institute of Oceanography, Ministry of Natural Resources, Hangzhou, 310012, China

^c^ School of Marine Sciences, Ningbo University, Ningbo, 315211, China

^d^ Key Laboratory of Applied Marine Biotechnology of Department of Education, Ningbo University, Ningbo, 315211, China

* For correspondence, E-mail: zhanghuajun@nbu.edu.cn (Huajun Zhang)

**Table S1** Genomic information of *Tamlana* sp. MS1

| **General features** | **MS1** |
| --- | --- |
| Genome Size(Mb) | 4.12 |
| GC content(%) | 42.17 |
| rRNA genes | 6 |
| tRNA genes | 40 |
| Coding density(%) | 90.76 |
| Protein coding sequences(CDS) | 3467 |
| Plasmid | 8 |

**Table S2** Comparison of digital DNA-DNA hybridization (dDDH) values between MS1 and other strains

| **Strain name** | ***Tamlana* sp. MS1(%)** |
| --- | --- |
| *Tamlana crocina* HST1-43 (GCA_012037625.1) | 19.4 |
| *Tamlana nanhaiensis* FHC16 (GCA_000943555.1) | 15.8 |
| *Tamlana sargassicola* 62-3 (GCA_020523965.1) | 14.6 |
| *Tamlana laminarinivorans* PT2-4 (GCA_020523925.1) | 14.6 |
| *Tamlana haliotis* B1N29 (GCA_008806375.1) | 14.6 |
| *Tamlana sedimentorum* JCM19808 (GCA_000943565.1) | 14.5 |
| *Tamlana fucoidanivorans* CW2-9 (GCA_006265225.1) | 14.5 |
| *Tamlana agarivorans* JW-26 (GCA_001642835.1) | 14.3 |
| *Tamlana carrageenivorans* UJ94 (GCA_002893765.1) | 14.2 |

**Table. S3** Predicted functional genes for *Tamlana* sp. MS1

| **Predicted function** | ***Tamlana* sp*. MS1*** |  |  |
| --- | --- | --- | --- |
| Vitamin biosynthesis or transport |  | Iron acquisition |  |
| biotin biosynthesis | + | iron hydroxamate transport |  |
| Vitamin B12 biosynthesis |  | iron ABC-type transport |  |
| Vitamin B12 transporters | 26 | FepD siderophore transport |  |
| folate biosynthesis | + | Bacterioferritin-associated ferrodoxin Bfd |  |
| thiamine biosynthesis | + | ferritin-like metal binding protein YciE |  |
| phylloquinone (Vitamin K1) biosynthesis | + | FebA/FepB siderophore transport | + |
| AA and Carbohydrate Transport/Metab. |  | Iron transporters FeoA/CirA |  |
| proteases | 11 | ferric dicitrate transport (FecR) |  |
| Spermidine*/putrescine* | 4 | Other predicted function |  |
| N-acetylglucosamine* | + | Plant Alkaloids | + |
| glycine | + | Pilin biosynthesis | + |
| cysteine | + | Capsule polysaccharide synthesis | + |
| glucose | + | gliding motility | + |
| Phenylacetate |  | swimming motility |  |
| pectate | + | bacteriophytochromes |  |
| fucose |  | PhyR (phyllosphere induced regulator) |  |
| glycerol | + | catalase | + |
| xylan | + | polyphosphate kinase | + |
| alginate | + |  |  |
| galactose | + |  |  |
| citrate | + |  |  |
| xylose | + |  |  |
| melibiose |  |  |  |
| mannitol/fructose |  |  |  |
| betaine |  |  |  |
| glycerol −3-phosphate | + |  |  |
| DMSP* demethylation |  |  |  |
| Sialic/Polysialic acid utilization | + |  |  |


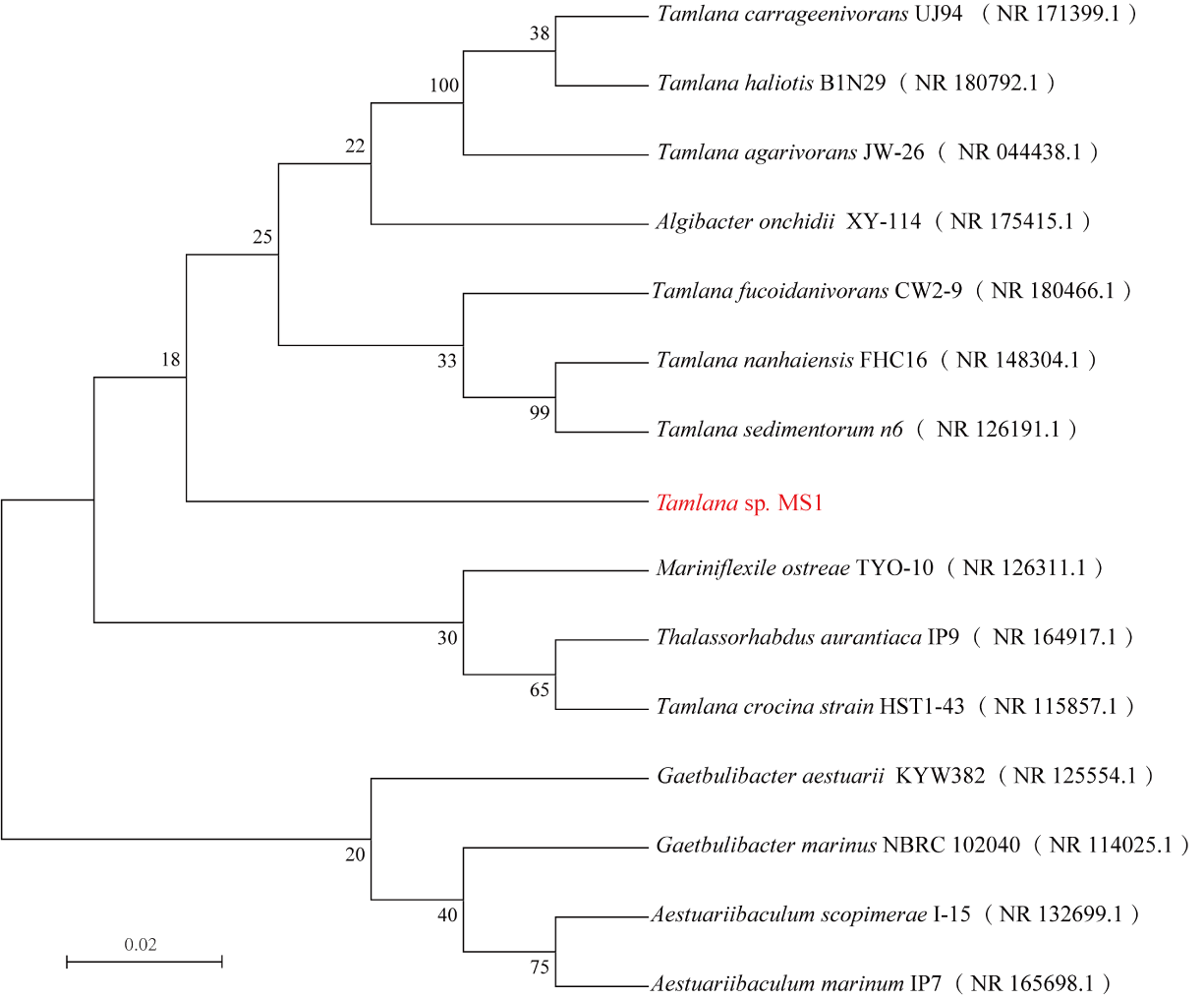


**Fig. S1** Phylogenetic tree of *Tamlana* sp. MS1 based on the 16S rDNA sequence.


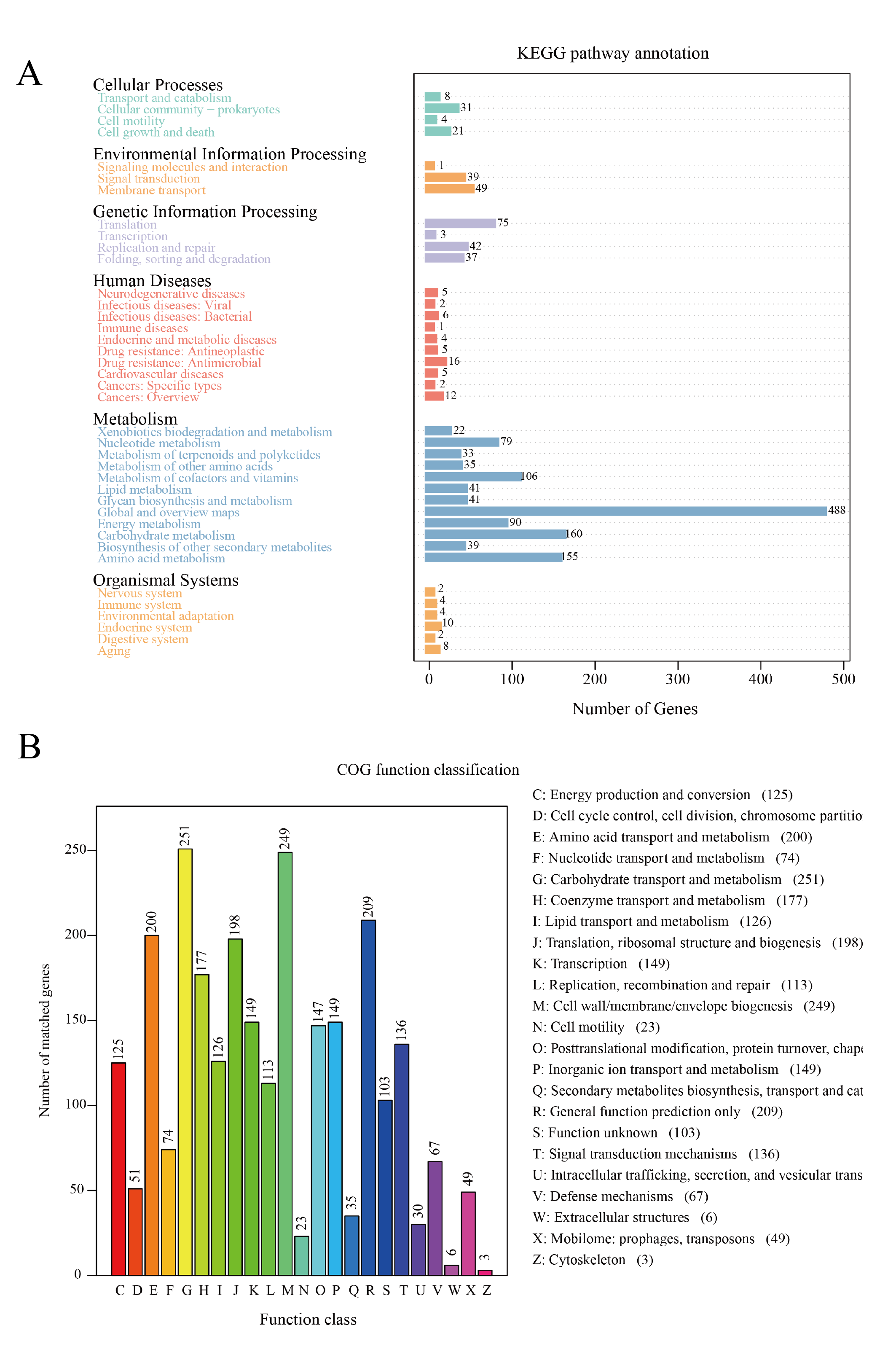


**Fig. S2** Gene annotation of MS1 by KEGG (A) and COG (B) database.


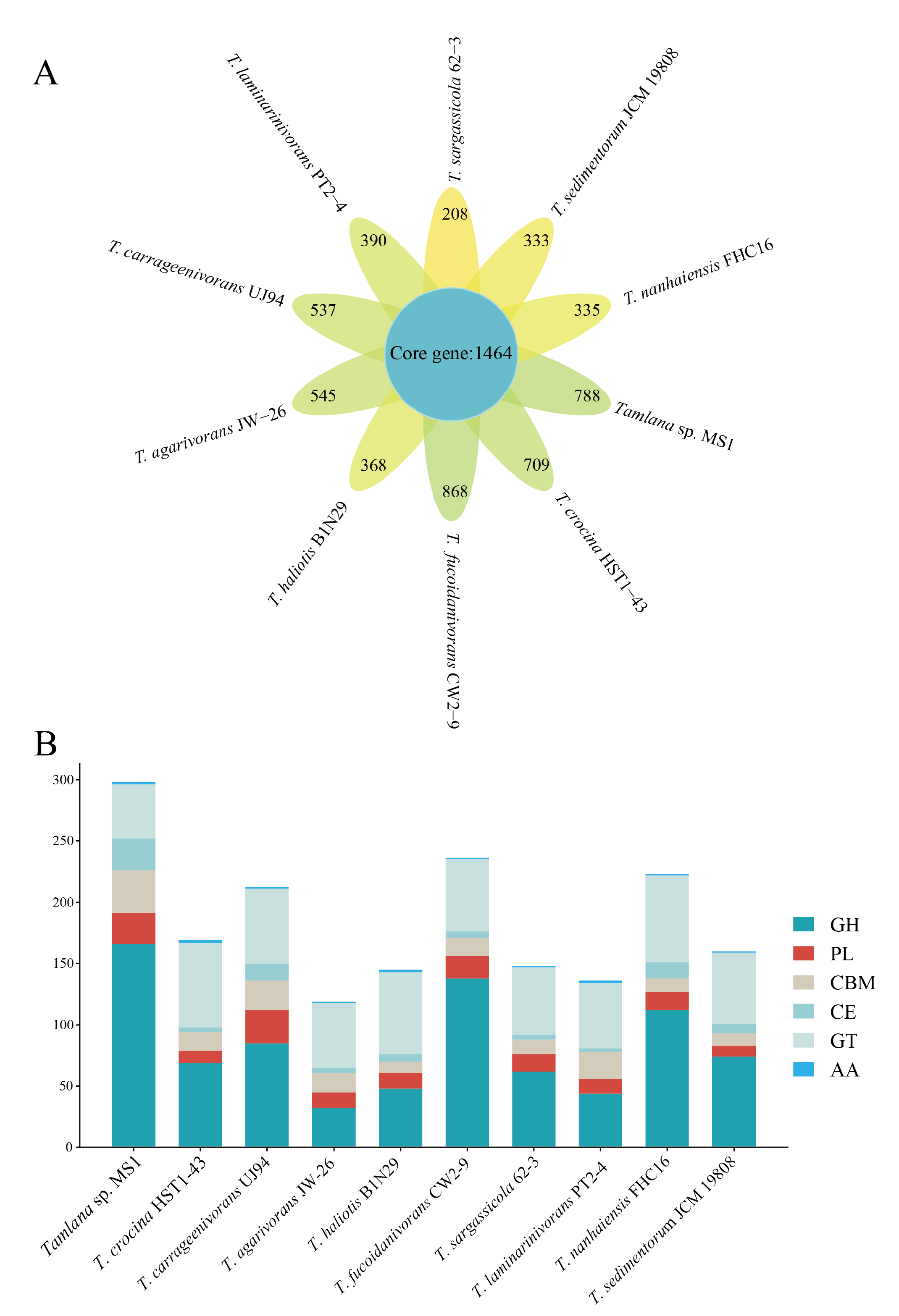


**Fig.S3** Comparative genomic analysis of *Tamlana* sp. MS1. A Pan-genome analysis of *Tamlana* sp. MS1 with 9 strains from the same genus. B: Genes of CAZyme family in *Tamlana* genus.
